# Simplified double barrel repair with autologous pericardium for tetralogy of fallot with hypoplastic pulmonary annulus and anomalous coronary crossing right ventricular outflow

**DOI:** 10.4103/0974-2069.41053

**Published:** 2008

**Authors:** Krishnanaik Shivaprakasha

**Affiliations:** Department of Pediatric Cardiac Surgery, Amrita institute of Medical Sciences and Research Center, Kochi, India

## INTRODUCTION

Variations in coronary artery anatomy are not uncommon and may complicate the surgical repair of Tetralogy of Fallot (TOF). In 5-9 % of the patients with TOF, a major coronary artery branch courses anteriorly across the right ventricular outflow tract (RVOT),[1–3] so that a transannular incision or patch would interrupt coronary blood flow. The most commonly encountered patterns are left anterior descending coronary artery (LAD) from right coronary artery (RCA), RCA from left coronary artery (LCA), RCA from LAD, large conal artery from RCA, and single RCA.[4] The immediate and long-term outlook depends on the adequate and sustained relief of RVOT obstruction without injury to a coronary artery supplying a significant portion of myocardium. Repair through the right atrium (RA), the right ventricle (RV) and main pulmonary artery (MPA) is possible only when the pulmonary annulus is considered adequate.[5] However, this is relatively uncommon. Varying degrees of hypoplasia of the pulmonary valve annulus and the main pulmonary artery (MPA) is frequently encountered. The most preferred surgical option in such a situation is placement of a conduit between RV and MPA for RVOT reconstruction.

In many centers in the developing world, conduits are unavailable or cost considerations do not permit their use. Further, most families cannot afford repeat operations for conduit changes. There have been several reports in the recent times that minimize the use of the conduits with alternate surgical techniques.[6–9] The “pulmonary-arterial-turn-back-technique” where the main pulmonary arterial flap is used to reconstruct the posterior wall of the new anterior outflow[4] cannot be applied to situations where the MPA is short or grossly hypoplastic. In this paper, a simplified “double -barrel” approach employing the same principle is described.

## THE SURGICAL TECHNIQUE

Selection criterion: All patients with diagnosis of TOF with anomalous coronary artery were taken up for surgery. Adequate intrapericardial pulmonary arterial branches was a prerequisite for total correction employing this technique. The adequacy of the artries was measured employing the standard nomograms.

**Figure 1:**
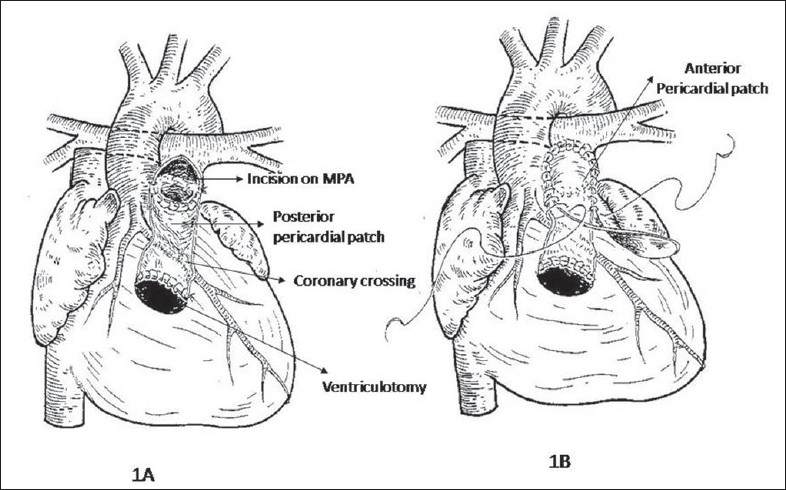
Drawing showing the creation of the double barrel. The posterior flap is shown in 1A. The anterior elliptical flap is shown in 1B

**Figure 2:**
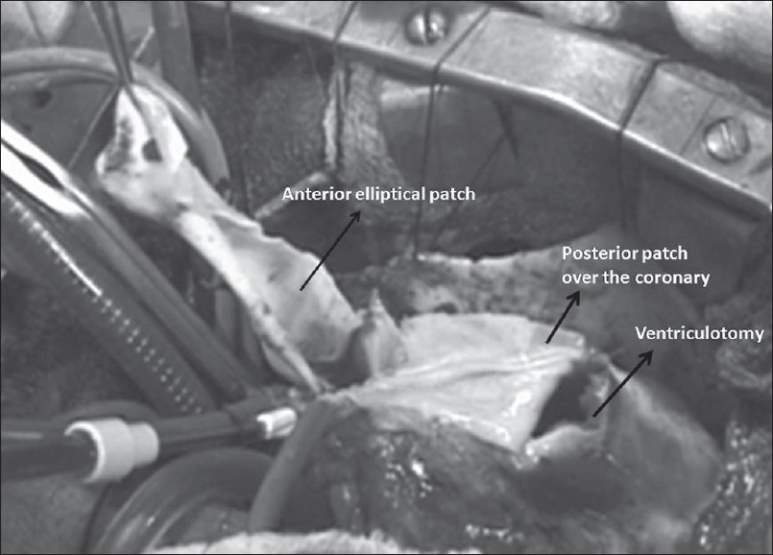
Operative photograph with the posterior flap in place and a part of the anterior flap sutured

**Figure 3:**
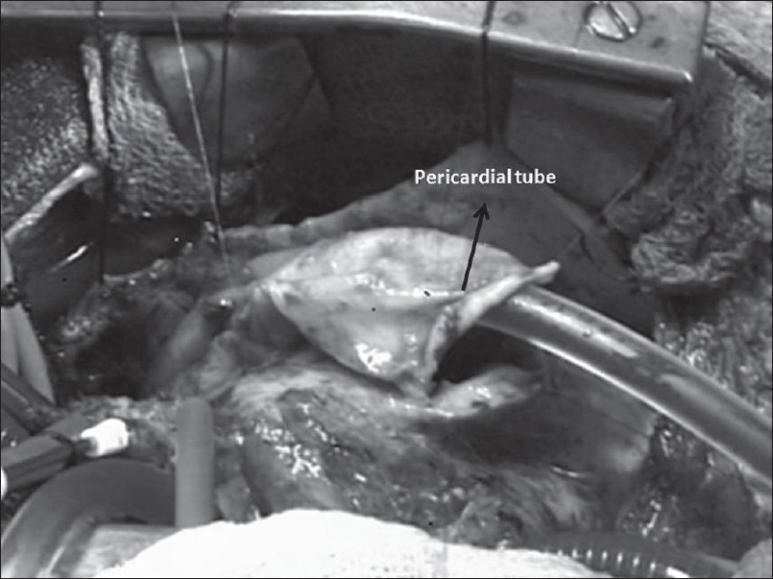
Operative photograph taken close to completing of the creation of the anterior barrel. A pump sucker is in situ inside the reconstructed anterior outflow.

**Figure 4:**
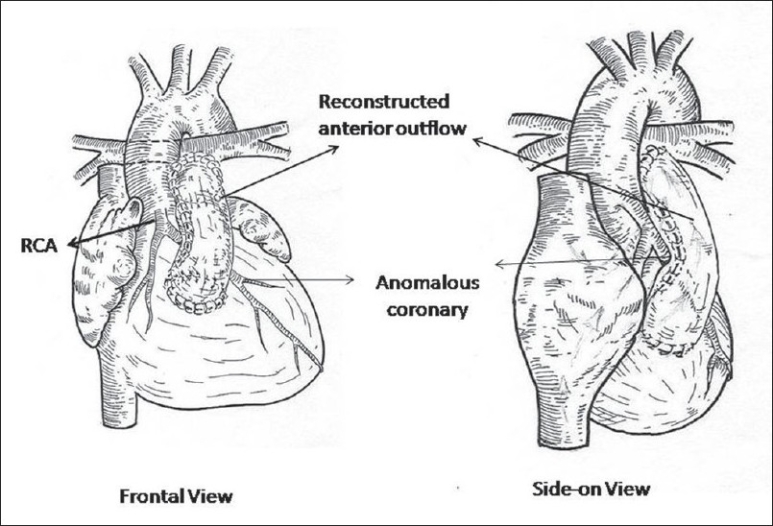
Drawing showing the completed procedure in frontal and “side-on” views

Moderate hypothermia, cardiopulmonary bypass and intermittent antegrade cardioplegia was used in all patients. The ventricular septal defect (VSD) was closed with poly-tetra-fluoro-ethylene (PTFE) patch employing continuous sutures from the right atrium. After infundibular resection from the atrial approach, the RVOT was opened below the anomalous vessel with an incision parallel to its course. The incision was oblique or horizontal depending upon the course of the anomalous coronary artery. The remaining hypertrophied muscle bundles within the RVOT were excised. Commissural fusion in the pulmonary valve was then released while ensuring the integrity of the valve. The MPA was opened up-to the bifurcation downstream to the annulus. An appropriate rectangular piece of the pericardium was then sutured between the superior edge of the RVOT incision and over the MPA at the annulus [Figure 1A]. The roof to the resultant RV-PA continuity was fashioned by employing an additional elliptical pericardial patch [Figures 1B, 2]. The anterior patch was 1 ½ times the width of the posterior pericardial patch. The resultant pericardial tube was adequate as predicted for the size of the patient to the maximum of 18 mm. This resulted in a double barrel outflow [Figures 3 and 4]. The posterior barrel is the native RVOT of the patient with the native pulmonary valve while the anterior barrel is the newly constructed RVOT. The anomalous coronary artery is located between the two “barrels” [Figure 4]. The meeting point of the suture lines were reinforced with pledgetted sutures thereby ensuring adequate hemostasis before discontinuing bypass. The rest of the procedure was done in the standard fashion.

### Results of the Procedure at our Institution

Sixteen patients with TOF with a small pulmonary annulus and anomalous coronary artery crossing the RVOT underwent total correction using double barrel technique from August 2002 to June 2006. The coronary artery was considered anomalous if its location interfered with the usual surgical repair of the obstructed RVOT and whose sacrifice would seriously compromise the viability of a significant portion of myocardium.[6] Records of all the 16 patients were reviewed and analyzed retrospectively. Age of the patients ranged from 6 months to fifteen years (mean 4.37 ± 3.81 yrs) at the time of operation. The mean weight was 13.35 ± 7.1 kg. Three of them had received systemic to pulmonary artery shunts and three underwent palliative balloon pulmonary valvotomy in infancy in view of poor oxygen saturation. Preoperatively, all the patients underwent thorough echocardiographic assessment of the anatomy. Cardiac catheterization was done in three patients for anatomic delineation of the collaterals and coronaries. All the patients had an anomalous coronary artery crossing the RVOT. The coronary anomalies consisted of the following: Dual LAD (n=7), LAD from RCA (n=4), large conal branch from LAD (n=3), RCA from LCA (n=1), single coronary artery arising from the left sinus with RCA crossing the RVOT (n=1). The operations were accomplished with a mean cardiopulmonary bypass time of 247 ± 69 min and cross clamp time of 154 ± 36 min.

Following the operation, the right ventricular to left ventricular pressure ratio was 0.65±0.13 measured directly at completion of the operation. Mean duration of inotropic supports and ICU stay were 2.67 ± 2.02 and 5.73 ± 2.37 days respectively. There was no early or late mortality.

Echocardiogram at discharge revealed good flows through both the outflow tracts in all the patients and one of them had significant RVOT gradient (41 mm Hg). The average hospital stay was eight days. The entire native outflow permitted antegrade flow without any regurgitation. The anteriorly reconstructed pericardial outflow demonstrated good antegrade flow and free regurgitation.

The follow-up period ranged from 15-70 months. One child had a small residual VSD identified on echo three months after surgery. It continues to be restrictive (less than 3 mm in size) for the last three years. However, he had three episodes of bacterial endocarditis that have been successfully treated. Three patients continued to require medications. Two patients (#1, #3) were receiving low doses (0.5 mg/kg/day) of frusemide and one required regular dose (1 mg/kg/day). Both outflows were patent and were demonstrating good antegrade flow in all patients at the time of last follow up.

## DISCUSSION

The anomalous coronary crossing the RVOT presents a unique problem among the patients with TOF and hypoplastic pulmonary annulus because it cannot be enlarged with the standard technique using a trans-annular patch. Several techniques have been advocated for circumventing this situation for achieving total correction.[4–7] A trans-atrial repair or a combination of trans-atrial and trans-pulmonary repair certainly satisfies those subsets where the annulus is adequate.[5] Surgical treatment continues to evolve for annular hypoplasia associated with anomalous coronary vessel.[4,6–9] Establishment of the RV-PA continuity with a conduit interposition remains a standard surgical therapy in these groups of patients.[10] However, the lack of availability and logistics of setting up of the homograft bank for the cryopreserved homografts continue to encourage the non-conduit reconstructions of the right ventricular outflow tracts.[11] Xenografts though offer off- the- shelf privileges, continue to be expensive. Additionally there is always the risk of subjecting the patient to many reoperations. Moreover, the short-term and the longt-term results of the hetero-homografts are not much different.[12–14] Our technique offers the advantage of one final surgery for these subsets. It avoids the cost of conduits during the total corrections thereby offering financial relief for the patients in our part of the world where public funding for cardiac surgery continues to be very low. Schlichter *et al* have demonstrated in their 15 year follow-up study of fresh autologus conduits with a excellent short and long-term outcome.[15] We share their belief that all these pericardial autologus conduits may not require any reoperations because the size of these conduits (16 mm and more).

The technique described by Von Son is an innovative technique that establishes the continuity by achieving an double outflow tract for right ventricle.[8] However this technique does not apply to those situations where the supra annular pulmonary hypoplasia exists. This is due to inadequacy of tissue available to create a flap of MPA in order to use it as a posterior layer. Our technique eliminated this variable by establishing a continuity by interposing a patch of autologous pericardium. We do agree that our modification may not allow for growth. Nonetheless we emphasize that the native outflow still has that potential and might grow. Our serial echocardiography in the long-term should answer that question. But short-term follow-up study (up to five years) is encouraging.

Pulmonary regurgitation that occurs through the anterior outflow is perhaps the most important limitation of this procedure just as it is for the trans-annular patch in most patients undergoing TOF repair. We acknowledge that the risk of pulmonary incompetence causing the right ventricular failure remains like in any other correction for TOF with transannular patch.

## CONCLUSIONS

Our experience demonstrates that TOF with anomalous coronary artery can be satisfactorily addressed with this technique with no additional mortality or morbidity. This is a simple and cost-effective alternative to conduits and may eliminate revisions. While short and intermediate term results are encouraging, long-term follow-up of this cohort is required to determine whether the right ventricular outflow remains unobstructed as the patient grows. The problems that accompany chronic pulmonary regurgitation in operated TOF are likely to remain after this operation.
